# Diabetes in Patients With Heart Failure With Reduced Ejection Fraction During Hospitalization: A Retrospective Observational Study

**DOI:** 10.3389/fendo.2021.727188

**Published:** 2021-08-12

**Authors:** Yiling Zhou, Miye Wang, Si Wang, Nan Li, Shengzhao Zhang, Siqi Tang, Qingyang Shi, Yuliang Zhao, Jingwen Li, Yuping Zeng, Huan Song, Haoming Tian, Shuangqing Li, Sheyu Li

**Affiliations:** ^1^Department of Endocrinology and Metabolism, West China Hospital, Sichuan University, Chengdu, China; ^2^The Informatic Center, West China Hospital, Sichuan University, Chengdu, China; ^3^Department of Cardiology, West China Hospital, Sichuan University, Chengdu, China; ^4^Department of Pharmacy, West China Hospital, Sichuan University, Chengdu, China; ^5^Chinese Evidence-based Medicine Center, Cochrane China Center and MAGIC China Center, West China Hospital, Sichuan University, Chengdu, China; ^6^Division of Nephrology, Kidney Research Institute, West China Hospital, Sichuan University, Chengdu, China; ^7^Department of Health Care Associated Infection Management, West China Hospital, Sichuan University, Chengdu, China; ^8^Department of Laboratory Medicine, West China Hospital, Sichuan University, Chengdu, China; ^9^West China Biomedical Big Data Center, West China Hospital, Sichuan University, Chengdu, China; ^10^Department of General Medicine, West China Hospital, Sichuan University, Chengdu, China

**Keywords:** HFrEF, diabetes, intubation, cardiogenic shock, acute kidney injury, length of stay, ICU, hospitalization

## Abstract

**Background:**

Diabetes is prevalent worldwide including hospitalized patients with heart failure with reduced ejection fraction (HFrEF). This retrospective study investigated the association of diabetes with in-hospital adverse events in patients with HFrEF.

**Methods:**

We analyzed data from electronic medical records of patients hospitalized with HFrEF in West China Hospital of Sichuan University from January 1, 2011, to September 30, 2018. Propensity score matching balances the baseline characteristics between patients with and without diabetes. Logistic and Poisson regressions investigated the association of diabetes with risks of intubation, cardiogenic shock, acute kidney injury (AKI), intensive care unit (ICU) admission and death during hospitalization, and length of ICU and hospital stay in the matched cases.

**Results:**

Among 6,022 eligible patients (including 1,998 with diabetes), 1,930 patient pairs with and without diabetes were included by propensity score matching. Patients with diabetes had a significantly increased risk of intubation (odds ratio [OR], 2.69; 95% confidence interval [CI], 2.25–3.22; *P*<0.001), cardiogenic shock (OR, 2.01; 95% CI, 1.72–2.35; *P*<0.001), AKI at any stage (OR, 1.67; 95% CI, 1.44–1.94; *P*<0.001), ICU admission (OR, 1.89; 95% CI, 1.65–2.15; *P*<0.001), and death (OR, 4.25; 95% CI, 3.06–6.02; *P*<0.001) during hospitalization. Patients with diabetes had longer ICU (median difference, 1.47 days; 95% CI, 0.96–2.08; *P*<0.001) and hospital stay (2.20 days; 95% CI, 1.43–2.86; *P*<0.001) than those without diabetes. There were potential subgroup effects by age and by hypertension, and CKD status on the association of diabetes with risk of AKI at any stage; and subgroup effects by sex and CKD status on the association of diabetes with risk of intubation. The increase in length of hospital stay was larger in patients without hypertension than those with hypertension.

**Conclusions:**

Among patients with HFrEF, those with diabetes have a worse prognosis, including a higher risk of in-hospital intubation, cardiogenic shock, AKI, ICU admission and death during hospitalization, and longer ICU and hospital stay.

## Introduction

Diabetes is a common comorbidity of heart failure (HF) and is present in more than 40% of patients with HF, representing an increasing disease burden worldwide (1–6). HF with reduced ejection fraction (HFrEF), defined as left ventricular ejection fraction (LVEF) <40%, is a common form of HF that can lead to disability and death in people with and without diabetes (5, 7). Diabetes leads to the development of HF through various mechanisms (7–13). Hyperglycemia-accelerated atherosclerosis, which is responsible for coronary artery disease, is the most common cause of HF in people living with type 2 diabetes (14, 15). Coronary artery stenosis leads to myocardial ischemia and possibly apoptosis or necrosis impairing the normal function of pump function. Diabetes in line with hypertension also attributes to myocardial remodeling in structure and function, and thus consequently abnormality of diastolic function, causing compensation and HF (14, 15). Other lesions that are highly related to diabetes myocardial fibrosis and microangiopathy, also injures the function of the heart, causing and exacerbating HF (8, 15, 16). Nevertheless, it remains unclear whether and how these pathophysiological changes of diabetes translate into the clinical practice in patients with HFrEF.

Controversial reports debate whether diabetes is associated with poorer in-hospital prognosis of hospitalized patients with HFrEF (17). Although some large registries presented diabetes was associated with higher in-hospital mortality (18, 19), while others did not identify any significant association (20, 21). Additionally, current related studies are only focused on in-hospital death, but not common nonfatal adverse events that are relevant to the specific organs of patients. To improve the in-hospital prognosis in this patient population, it is critical to identify the impact of diabetes on specific patient-important clinical outcomes before the occurrence of death. We thus investigated the association of diabetes with in-hospital, clinically relevant respiratory, cardiovascular, and kidney system events, including intubation, cardiogenic shock, acute kidney injury (AKI), intensive care unit (ICU) admission and death during hospitalization, and length of stay in ICU and hospital, to comprehensively evaluate the effect of diabetes on HFrEF prognosis.

## Methods

### Data Source and Study Population

This retrospective cohort study enrolled Chinese patients hospitalized with HFrEF in the West China Hospital of Sichuan University between January 1, 2011, and September 30, 2018, based on electronic medical records (EMRs) which could be accessed *via* the Clinical Research and Exploration System of West China Hospital, Sichuan University. We included patients 1) aged ≥18 years; 2) with a length of hospitalization stay >2 days; 3) with all echocardiography LVEF values <40% during hospitalization; 4) with available records on prescription and discharge diagnosis; and 5) with available laboratory data (serum creatinine, serum alanine aminotransferase [ALT], triglyceride, total cholesterol [TC], low-density lipoprotein [LDL-c], high-density lipoprotein [HDL-c], blood glucose, and hemoglobin) and vital signs (heart rate, and systolic and diastolic blood pressure [BP]).

### Data Collection

We collected the following data for each patient from EMRs, including age, sex, smoking status, alcohol consumption, discharge diagnosis with International Classification of Diseases, 10th Revision (ICD-10) codes, admission department (Department of Cardiology/others), admission date, calendar year of admission date, discharge date, and patient status on discharge. We also collected prescription information **(** **Supplementary Table 1** **)**, the dates and records of resuscitation from the prescription records, vital signs (systolic and diastolic BP, heart rate, and respiration rate), height, and weight from the nursing records, laboratory test results from the laboratory information records **(** **Supplementary Table 1** **)**, and LVEF from the echocardiography reading. For patients with more than one hospitalization, we extracted the data pertaining to the last hospitalization. Where multiple values were available for a given parameter at the hospitalization, we used the earliest value to describe the characteristics of patients. The estimated glomerular rate filtration (eGFR) was calculated according to the chronic kidney disease epidemiology collaboration (CKD-EPI) formula (22). The Charlson Comorbidity Index (CCI) calculation (23, 24) was used to calculate an adjusted CCI that excluded myocardial infarction, congestive heart failure, and diabetes without/with chronic complication scores based on discharge diagnosis ICD-10 codes; the adjusted CCI was used to evaluate patient comorbidities. The ICD-10 codes for other comorbidities are summarized in **Supplementary Table 2**. All missing and abnormal values are identified as not available.

### Exposure

Patients were identified as having diabetes if they had at least one laboratory record of fasting glucose >7.0 mmol/L, 2-hour blood glucose after 75 g glucose challenge >11.1 mmol/L, random glucose >11.1 mmol/L, or glycated hemoglobin A1c (HbA1c) >6.5% during hospitalization, or a discharge diagnosis of ICD-10 codes E10–E14 (25). Others were recognized as without diabetes.

### Adverse Events

The following adverse events during hospitalization were identified:

- intubation: if a patient required an invasive ventilator.
- cardiogenic shock: if a patient required positive inotropic drugs such as intravenous milrinone, dobutamine, or noradrenaline for emergency use.
- AKI: identified adopting from a previous report (26) with their severity (stage 1–3) based on the Kidney Disease: Improving Global Outcomes guideline (27). Our endpoints included AKI at any stage, and we further analyzed AKI stage 2 or stage 3, and AKI stage 3 which is of the most severe as additional endpoints.
- ICU admission: if a patient was admitted to the medical ICU.
- death: if there was a death record or resuscitation records within one calendar day before voluntary discharge.
- length of stay in ICU: the total days of a patient treated in medical ICU.
- length of stay in hospital: the total days of a patient treated in the hospital.

### Follow-Up

The follow-up time started on the date of admission to hospital and ended on when the date of a given outcome or discharge comes first.

### Statistical Analysis

Baseline characteristics are presented using summary statistics, including mean ± standard deviation (SD) for continuous variables with normal distribution by the Kolmogorov–Smirnov test (P ≥0.001), and median (25% quantile, 75% quantile) if not normal, compared between groups using the two-sided Student’s *t*-test or the Mann–Whitney U test. Categorical variables were described as frequencies (percentages) and compared using the Chi-square test.

Propensity scores (PS) were used to estimate the probability of having diabetes using a multivariable logistic regression model, conditional upon age, sex, heart rate, eGFR, +/− ischemic heart disease (IHD), systolic BP, LVEF, CCI, calendar year of admission date, and admission department (Department of Cardiology versus others) (28). Each patient with diabetes was PS-matched 1:1 with a patient without diabetes. The nearest neighbor matching algorithm with a caliper of 0.02 was applied (29). We presented between-group differences in covariates before and after matching by the overlap in PS between two groups **(** **Supplementary Figure 1** **)**. The standardized differences in covariates between groups before and after PS matching (PSM) were calculated, and covariates with a standardized difference >0.10 after matching were considered sub-optimally matched.

We estimated the average treatment effect on the treated (ATT) based on each PS-matched sample. We assessed the association of diabetes with each dichotomous adverse event during hospitalization using Logistic regression and reported odds ratios (ORs) and 95% confidence intervals (CIs). We applied generalized Poisson regressions to estimate the time ratio of the length of ICU stay and hospitalization stay between patients with and without diabetes, and then estimated the excess length of ICU stay and hospital stay as well as its 95% CI in patients with diabetes. We also examined the impact of diabetes on in-hospital adverse events in subgroups stratified by sex, age, +/− HD, +/− hypertension, and +/− CKD, which was defined as eGFR <60 mL/min/1.73 m^2^. We calculated the E-value to quantify the minimum magnitude of potential unmeasured confounders that could explain the observed association between diabetes and each clinical outcome (30).

All analyses were conducted using R Studio (R Pack Version 3.6.1, R Studio, R Core Team, 2019, Boston, MA, USA) (31) and figures were produced using packages of *ggplot2* (Wickham, 2016) (32) and *forestplot* (Max Gordon and Thomas Lumley, 2020) (33). A two-sided *P* value <0.05 was considered statistically significant.

## Results

**Figure 1:**
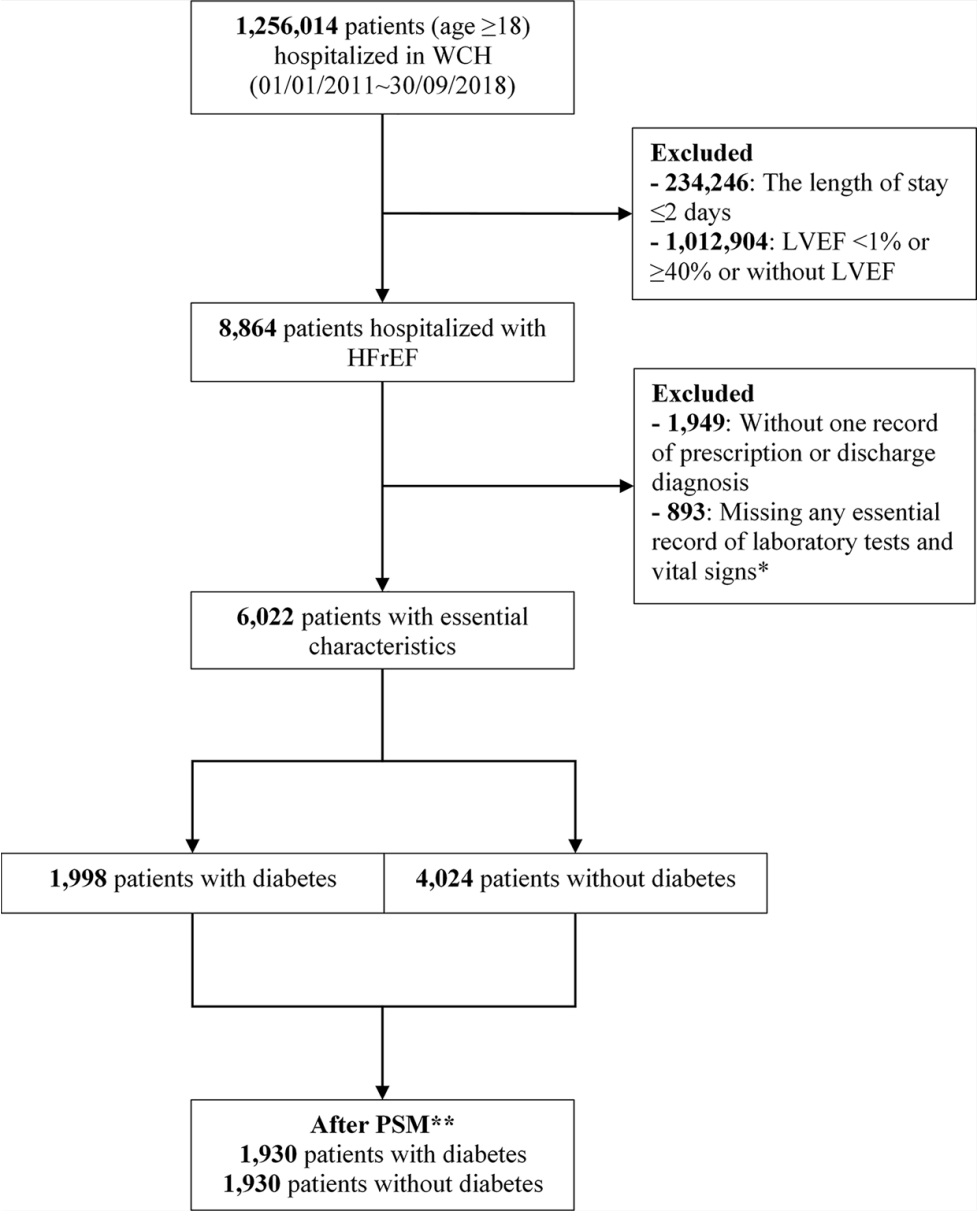
Flowchart of study population selection. *Essential records of laboratory tests included serum creatinine, serum alanine aminotransferase, triglyceride, total cholesterol, low-density lipoprotein, high-density lipoprotein, blood glucose, and hemoglobin, and vital signs included heart rate, and systolic and diastolic blood pressure. **Propensity score matching with ratio=1, caliper=0.02, adjusted for age, sex, heart rate, estimated glomerular filtration rate, +/− ischemic heart disease, systolic blood pressure, LVEF, Charlson Comorbidity Index, calendar year of admission date, and admission department. LVEF, left ventricular ejection fraction; PSM, propensity score matching; WCH, West China Hospital.

As shown in **Figure 1**, we included 6,022 patients (1,998 [33.2%] patients with diabetes) from 8,864 patients hospitalized with HFrEF, of whom 1,930 patient pairs with and without diabetes were PS-matched. Before matching, patients with diabetes were significantly older than those without (64.1 ± 13.2 years vs 59.4 ± 15.6 years; *P*<0.001) and were more likely to have IHD (51.7% [n=1,033] vs 32.1% [n=1,293]; *P*<0.001). Compared with patients without diabetes, those with diabetes had a significantly higher median (*P*<0.001) N-terminal pro-B type natriuretic peptide (NT-proBNP; 4,411.0 pg/mL vs 3,757.5 pg/mL), blood glucose (7.7 mmol/L vs 5.4 mmol/L), HbA1c (7.2 mmol/L vs 5.8 mmol/L), and CCI (1.0 vs 0.0), but lower eGFR (65.9 mL/min/1.73 m^2^ vs 76.9 mL/min/1.73 m^2^; *P*<0.001) **(** **Table 1** **)**. The overlap in PS between groups pre- and post-PSM is shown in **Supplementary Figure 1**. Covariates were balanced between groups after PSM **(** **Supplementary Table 3** **)**.

**Table 1:** Baseline patient demographic and clinical characteristics.

Characteristics	Total	Non-diabetes	Diabetes	P value
N, n (%)	6,022	4,024 (66.8)	1,998 (33.2)	
Age, years	61.0 ± 15.0	59.4 ± 15.6	64.1 ± 13.2	<0.001
Sex, female, n (%)	1721 (28.6)	1179 (29.3)	542 (27.1)	0.08
Alcohol use, n (%)	2097 (35.6)	1404 (35.6)	693 (35.5)	0.95
Smoking, n (%)	3538 (59.8)	2355 (59.5)	1183 (60.2)	0.64
Admission department, cardiology, n (%)	3367 (55.9)	2258 (56.1)	1109 (55.5)	0.67
LVEF, %*	33.0 (28.0, 36.0)	33.0 (28.0, 36.0)	33.0 (28.0, 36.0)	0.49
Heart rate, beats/minute*	82.0 (72.0, 98.0)	82.0 (72.0, 97.0)	84.0 (74.0, 98.0)	0.001
Systolic BP, mmHg*	120.0 (106.0, 134.0)	119.0 (106.0, 133.0)	120.0 (108.0, 136.0)	<0.001
Diastolic BP, mmHg*	73.0 (64.0, 84.0)	74.0 (64.0, 84.0)	73.0 (64.0, 82.0)	0.13
TC, mmol/L*	3.8 (3.1, 4.6)	3.9 (3.2, 4.6)	3.7 (3.0, 4.6)	<0.001
LDL-c, mmol/L*	2.2 (1.6, 2.8)	2.2 (1.7, 2.8)	2.1 (1.5, 2.7)	<0.001
TG, mmol/L*	1.1 (0.9, 1.6)	1.1 (0.8, 1.5)	1.2 (0.9, 1.7)	<0.001
HDL-c, mmol/L*	1.1 (0.8, 1.4)	1.1 (0.8, 1.4)	1.0 (0.8, 1.3)	<0.001
HbA1c (%)*	6.5 (5.9, 7.5)	5.8 (5.5, 6.1)	7.2 (6.6, 8.3)	<0.001
Blood glucose, mmol/L*	5.8 (5.0, 7.5)	5.4 (4.8, 6.4)	7.7 (5.9, 10.9)	<0.001
NT-proBNP, pg/mL*	3921.0 (1629.0, 9141.8)	3757.5 (1571.8, 8579.2)	4411.0 (1756.8, 9938.5)	<0.001
Hb, g/L*	131.0 (115.0, 145.0)	132.0 (116.0, 146.0)	129.0 (110.0, 142.0)	<0.001
ALT, mU/L*	25.0 (16.0, 46.0)	25.0 (16.0, 45.0)	26.0 (16.0, 48.0)	0.06
eGFR, mL/min/1.73 m^2^*	73.6 (50.4, 92.2)	76.9 (55.6, 94.3)	65.9 (43.0, 86.8)	<0.001
IHD, n (%)	2326 (38.6)	1293 (32.1)	1033 (51.7)	<0.001
Comorbidities				
CCI*	0.0 (0.0, 1.0)	0.0 (0.0, 1.0)	1.0 (0.0, 2.0)	<0.001
Hypertension, n (%)	2256 (37.5)	1298 (32.3)	958 (47.9)	<0.001
Arrhythmia, n (%)	2209 (36.7)	1498 (37.2)	711 (35.6)	0.22
Cardiomyopathy, n (%)	1492 (24.8)	1145 (28.5)	347 (17.4)	<0.001
RHD, n (%)	578 (9.6)	415 (10.3)	163 (8.2)	0.009
AMI, n (%)	727 (12.1)	361 (9.0)	366 (18.3)	<0.001

*Data are presented as median (interquartile range) unless otherwise stated.

ALT, alanine aminotransferase; Hb, hemoglobin; NT-proBNP, N-terminal pro-B-type natriuretic peptide; CCI, Charlson Comorbidity Index; eGFR, estimated glomerular filtration rate; LDL-c, low-density lipoprotein; LVEF, left ventricular ejection fraction; BP, blood pressure; IHD, ischemic heart disease; TC, total cholesterol; HDL-c, high-density lipoprotein; TG, triglyceride; RHD, rheumatic heart disease; AMI, Acute myocardial infarction; HbA1c, hemoglobin A1c.

**Figure 2:**
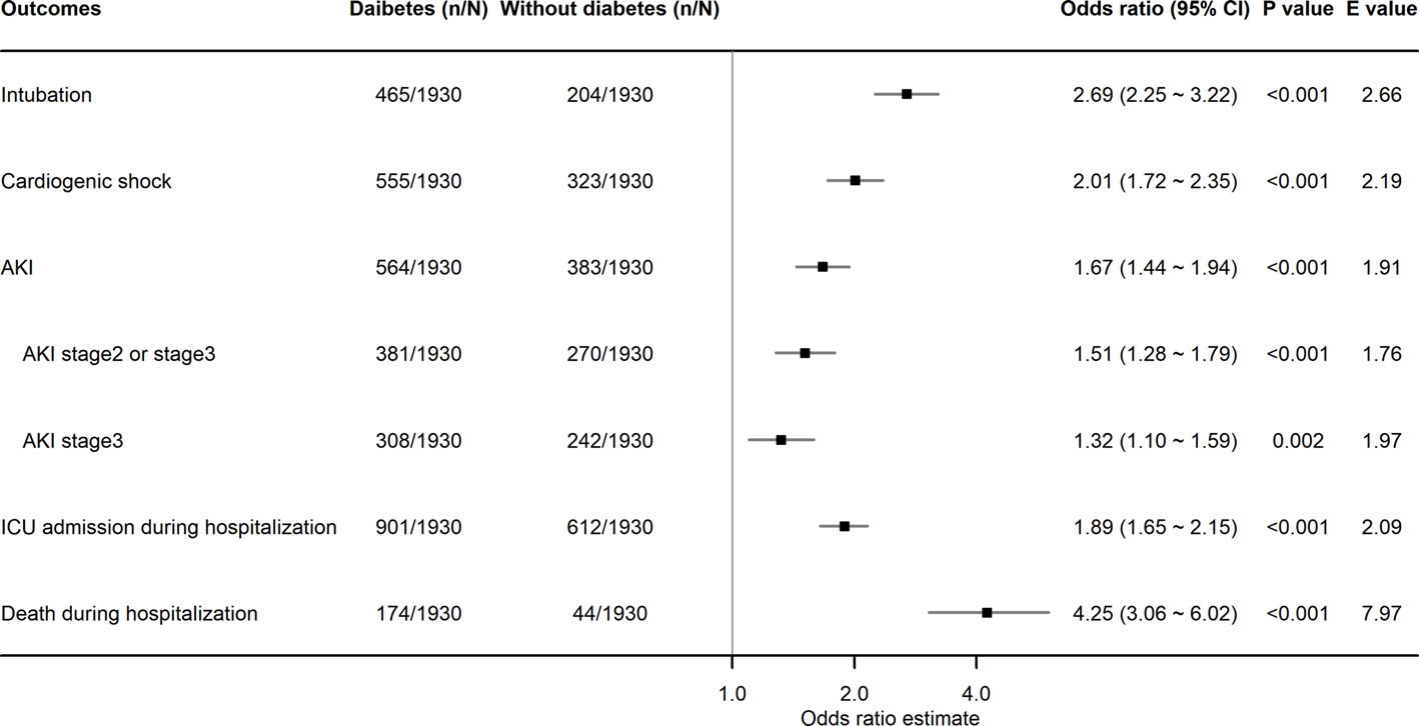
Association of diabetes with binary in-hospital adverse events in patients hospitalized with HFrEF. HFrEF, heart failure with reduced ejection fraction; AKI, acute kidney injury; ICU, intensive care unit.

**Figure 3:**
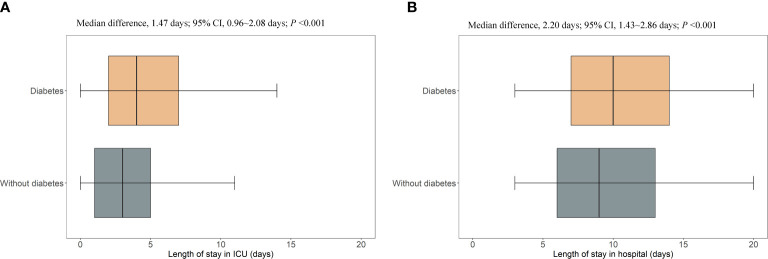
Excess length of ICU stay and hospital stay in patients with diabetes who were hospitalized with HFrEF. ICU, intensive care unit; CI, confidence interval. **(A)**, the length of stay in intensive care unit; **(B)**, the length of stay in hospital.

After PSM, diabetes was found to be significantly associated with higher risks of intubation (OR, 2.69; 95% CI, 2.25–3.22; *P*<0.001), cardiogenic shock (OR, 2.01; 95% CI, 1.72–2.35; *P*<0.001), AKI in any stage (OR, 1.67; 95% CI, 1.44–1.94; *P*<0.001), ICU admission (OR, 1.89; 95% CI, 1.65–2.15; *P*<0.001), and death (OR, 4.25; 95% CI, 3.06–6.02; *P*<0.001) during hospitalization. Patients with diabetes also had a higher incidence risk of AKI stage 2 or 3 (OR, 1.51; 95% CI, 1.28–1.79; *P*<0.001) and AKI stage 3 (OR, 1.32; 95% CI, 1.10–1.59; *P*=0.002) during hospitalization **(** **Figure 2** **)**. The observed associations between diabetes and the outcomes of intubation, cardiogenic shock, AKI in any stage, AKI stage 2 or stage 3, AKI stage 3, ICU admission, and death could be explained by the existence of unmeasured confounding, with ORs over 2.66, 2.19, 1.91, 1.76, 1.97, 2.09, and 7.97, respectively **(** **Figure 2** **)**. The excess median length of ICU and hospital stay in patients with diabetes was 1.47 days (95% CI, 0.96–2.08; *P*<0.001) and 2.20 days (95% CI, 1.43–2.86; *P*<0.001), respectively **(** **Figure 3** **)**.

**Figure 4:**
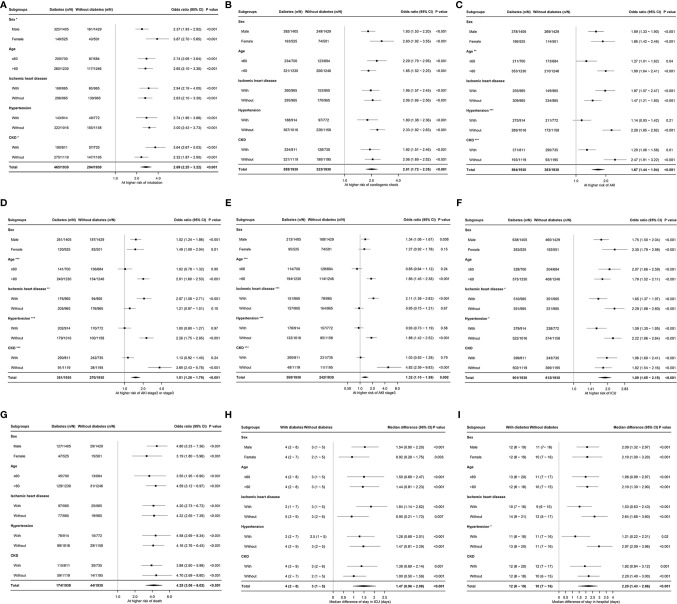
Association of diabetes and the risks of in-hospital adverse events in patients hospitalized with heart failure with reduced ejection fraction. *P interaction <0.05; **P interaction <0.01; ***P interaction <0.001. CKD, chronic kidney disease; AKI, acute kidney injury; ICU, intensive care unit; CI, confidence interval. **(A)**, risk of intubation; **(B)**, risk of cardiogenic shock; **(C)**, risk of AKI; **(D)**, risk of AKI stage 2 or stage 3; **(E)**, risk of AKI stage 3; **(F)**, risk of ICU admission; **(G)**, risk of death during hospitalization; **(H)**, the length of stay in intensive care unit; **(I)**, the length of stay in hospital.

**Figures 4A–I** presented the results of subgroup analyses of each adverse event. As shown in **Figure 4C**, subgroup analyses indicated a differential impact of diabetes on the risk of incident AKI in any stage, with a stronger association in patients aged >60 years (vs aged ≤60 years, interaction *P*<0.01), without hypertension (vs with hypertension, interaction *P*<0.001), or without CKD (vs with CKD, interaction *P*<0.001). Similar subgroup effects by age, +/− hypertension, and +/− CKD were found for the association of diabetes with risks of AKI stage 2 or stage 3 and AKI stage 3, as well as the subgroup effect by +/− IHD, shown in **Figures 4D, E**. A significant interaction between diabetes and hypertension or IHD was found in the analysis of ICU admission, in that the association of diabetes with a higher risk of ICU admission during hospitalization was stronger in patients without hypertension (vs with hypertension, interaction *P*<0.05) or without IHD (vs with IHD, interaction *P*<0.05), shown in **Figure 4F**. Diabetes significantly increased the length of hospital stay in patients without hypertension, but not in patients with hypertension (interaction *P*<0.001), shown in **Figure 4I**.

## Discussion

In the present study, approximately one-third of patients hospitalized with HFrEF had diabetes, and diabetes had a negative impact on short-term prognosis before discharge, increasing the risks of adverse events of respiratory, cardiovascular, kidney systems as well as the ICU admission and death during hospitalization, and extending the length of ICU and hospital stays. Our findings indicate there is a clinical need for increased focus on the respiratory, cardiovascular, and kidney systems of patients with both diabetes and HFrEF during hospitalization and for appropriate management to prevent in-hospital death.

In the present study, patients hospitalized with HFrEF who also had diabetes experienced longer hospitalization stay and ICU stay as well as a higher admission rate to ICU, in line with previous findings (4, 17, 21, 34, 35), which indicated that diabetes could complicate the clinical status of patients with HFrEF. Although the findings of previous studies on the impact of diabetes on in-hospital death in patients with HF remain controversial (18–21), our findings support the suggestion that diabetes increases in-hospital death in patients with HF (18, 19) but stand in contrast to the outcomes of the OPTIMIZE-HF study (20), potentially because of heterogeneity in the medication regimen of patients in that study, who received aggressive evidence-based management, suggesting the need to establish appropriate management based on recent evidence. The shift of therapeutic technology and prevalence of diabetes in patients with HFrEF explained the difference in findings between our study and that of van den Berge et al. (21). Without adjusting calendar year in cohort studies with a long-time span may result in false-negative association with the increasing incidence of diabetes and improved prognosis of HF. Our study balanced covariates including calendar-year strata and found that diabetes linked to excessive in-hospital death and other adverse events, calling for attention and possibly additional care to diabetes-comorbid HFrEF patients during hospitalization.

Our findings also expand the understanding of the impact of diabetes on short-term adverse clinical outcomes in patients hospitalized with HFrEF. We observed a higher rate of AKI among HFrEF patients with diabetes, consistent with previous findings (36–39). It indicates that the kidney in patients with diabetes may be more “fragile” due to accumulated lesions caused by diabetes, especially in the attack of HF (39). Dehydration and the use of anti-diabetic drugs may also contribute as one of the risk factors. It calls for intensive monitoring of the volume and glucose status in patients with diabetes and careful prescription of anti-diabetic drugs (40). For example, clinicians may consider stopping metformin before contrast-based CT scans or interventional therapy (41). To be noted, the volume response is complicated and further action to protect the kidney is necessary in people with HFrEF (42).

Strengths of our study included the selection of patients hospitalized with HFrEF as the study population, including those in ICUs, which enabled investigation of the association of diabetes with a short-term prognosis of HFrEF. Second, we analyzed five patient-important outcomes covering cardiovascular, kidney, and respiratory systems to comprehensively evaluate the clinical prognosis of HFrEF. Furthermore, we adjusted for the calendar year to improve the covariate balance when applying PSM, which enhanced the power of our study. We calculated the E-value of the primary analyses to evaluate the strength of the association between diabetes and each adverse event. Our study also had some limitations, including the single-center design and the inability to conclude any causation between diabetes and the adverse outcomes.

## Conclusions

The short-term prognosis for patients with HFrEF is worse in those with diabetes as comorbidity, with this population being more likely to receive intubation, develop new-onset AKI or cardiogenic shock, be admitted to ICU, die during hospitalization, and have a longer ICU stay or hospital stay. Clinicians need to monitor the patient’s condition more closely in those with both diabetes and HFrEF, who may face additional in-hospital risks than those without diabetes.

## Data Availability Statement

The datasets presented in this article are not readily available because the individual-level data was restricted in storage and utilized within the informatic server of West China Hospital. Any public use and transaction were prohibited to protect the privacy of the patients. Requests to access the datasets should be directed to Lisheyu@gmail.com.

## Ethics Statement

This study was approved by the ethics committee of West China Hospital, Sichuan University (No. 2019-472). Patient consent was waived for this retrospective study of data from electronic medical records.

## Author Contributions 

All authors made substantial contributions to the conception and design of the work, the acquisition, analysis, and interpretation of data, and the drafting or critical revision of the article for important intellectual content. All authors contributed to the article and approved the submitted version.

## Funding

SYL received grants from the Sichuan Science and Technology Program (grant number 2019YFH0150) and the 1.3.5 Project for Disciplines of Excellence, West China Hospital, Sichuan University (grant number ZYGD18022 and 2020HXF011), National Natural Science Foundation of China (grant number 21534008), and the Chief Scientist Office Project (reference number CGA/19/10).

## Conflict of Interest

The authors declare that the research was conducted in the absence of any commercial or financial relationships that could be construed as a potential conflict of interest.

## Publisher’s Note

All claims expressed in this article are solely those of the authors and do not necessarily represent those of their affiliated organizations, or those of the publisher, the editors and the reviewers. Any product that may be evaluated in this article, or claim that may be made by its manufacturer, is not guaranteed or endorsed by the publisher.

## Abbreviations

HF, heart failure; HFrEF, heart failure with reduced ejection fraction; AKI, acute kidney injury; ICU, intensive care unit; EMRs, electronic medical records; ICD-10, International Classification of Diseases, 10th Revision; CI, confidence interval; OR, odds ratio; BP, blood pressure; HR, heart rate; LVEF, left ventricular ejection fraction; CCI, Charlson Comorbidity Index; IHD, ischemic heart disease; PS, propensity score; PSM, propensity score matching; ALT, alanine aminotransferase; Hb, hemoglobin; NT-proBNP, N-terminal pro-B-type natriuretic peptide; eGFR, estimated glomerular filtration rate; LDL-c, low-density lipoprotein; LVEF, left ventricular ejection fraction; TC: total cholesterol; HDL-c: high-density lipoprotein; TG: triglyceride; RHD, rheumatic heart disease; AMI, acute myocardial infarction; HbA1c, hemoglobin A1c.
