# Supplementary material for: Diabetes in Patients With Heart Failure With Reduced Ejection Fraction During Hospitalization: A Retrospective Observational Study
Source: Front Endocrinol (Lausanne). 2021 Aug 12;12:727188. doi: 10.3389/fendo.2021.727188 (PMC8387582; doi:10.3389/fendo.2021.727188)
Supplement: Supplementary file 1 [file DataSheet_1.docx]

Supplementary Material

# Supplementary Figures and Tables

## Supplementary Tables

**Supplementary table 1:** The details of prescription information and laboratory tests.

| **Prescription information** | **Laboratory tests** |
| --- | --- |
| - Resuscitation - Intubation - Intravenous milrinone - Dobutamine - Noradrenaline | - Alanine aminotransferase - Hemoglobin - N-terminal pro-B-type natriuretic peptide - Serum creatinine - Low-density lipoprotein - Total cholesterol - High-density lipoprotein - Triglyceride - Blood glucose - Fasting glucose - 2-hour blood glucose after 75 g glucose challenge - Random glucose - Glycated Hemoglobin A1c |

**Supplementary table 2:** The international Classification of Diseases, 10th Revision (ICD-10) codes for identifying other comorbidities from discharge diagnosis records.

| **Comorbidities** | **ICD-10 codes** |
| --- | --- |
| Hypertension | I10 to I15 |
| Arrhythmia | I44 to I49 |
| Cardiomyopathy | I42 |
| Rheumatic heart disease | I01 to I09 |
| Acute myocardial infarction | I21 to I23 |
| Ischemic heart disease | I20 to I25 |

| Characteristics | Before | | | | |  | After | | | | |
| --- | --- | --- | --- | --- | --- | --- | --- | --- | --- | --- | --- |
|  | Total | Patients without diabetes | Patients with diabetes | Standardized difference | P value |  | Total | Patients without diabetes | Patients with diabetes | Standardized difference | P value |
| N (%) | 6022 | 4024 | 1998 |  |  |  | 3862 | 1931 | 1931 |  |  |
| Age, years | 61.0±15.0 | 59.4±15.6 | 64.1±13.2 | 0.32 | <0.001 |  | 63.9±13.8 | 64.0±14.5 | 63.7±13.1 | 0.02 | 0.46 |
| LVEF (%) * | 33.0 [28.0, 36.0] | 33.0 [28.0, 36.0] | 33.0 [28.0, 36.0] | 0.02 | 0.49 |  | 33.0 [28.0, 36.0] | 32.0 [28.0, 36.0] | 33.0 [28.0, 36.0] | 0.006 | 0.81 |
| eGFR, mL/min/1.73 m^2^ * | 73.6 [50.4, 92.2] | 76.9 [55.6, 94.3] | 65.9 [43.0, 86.8] | 0.30 | <0.001 |  | 67.9 [45.2, 87.4] | 68.8 [46.7, 87.2] | 66.9 [44.4, 87.9] | 0.01 | 0.50 |
| HR, beats/minute * | 82.0 [72.0, 98.0] | 82.0 [72.0, 97.0] | 84.0 [74.0, 98.0] | 0.09 | <0.001 |  | 83.0 [74.0, 98.0] | 83.0 [73.0, 99.0] | 84.0 [74.0, 98.0] | 0.002 | 0.72 |
| Systolic BP, mmHg * | 120.0 [106.0, 134.0] | 119.0 [106.0, 133.0] | 120.0 [108.0, 136.0] | 0.12 | <0.001 |  | 120.0 [108.0, 135.0] | 120.0 [108.0, 135.0] | 120.0 [108.0, 135.0] | 0.01 | 0.80 |
| CCI * | 0.0 [0.0, 1.0] | 0.0 [0.0, 1.0] | 1.0 [0.0, 2.0] | 0.13 | <0.001 |  | 1.0 [0.0, 2.0] | 1.0 [0.0, 2.0] | 1.0 [0.0, 2.0] | 0.008 | 0.99 |
| Female (%) | 1721 (28.6) | 1179 (29.3) | 542 (27.1) | 0.05 | 0.08 |  | 1055 (27.3) | 527 (27.3) | 528 (27.3) | 0.001 | >0.99 |
| IHD (%) | 2326 (38.6) | 1293 (32.1) | 1033 (51.7) | 0.40 | <0.001 |  | 1927 (49.9) | 960 (49.7) | 967 (50.1) | 0.007 | 0.85 |
| Admission department, Cardiology (%) | 3367 (55.9) | 2258 (56.1) | 1109 (55.5) | 0.01 | 0.67 |  | 2154 (55.8) | 1076 (55.7) | 1078 (55.8) | 0.002 | 0.97 |
| Calendar year of admission date (%) |  |  |  |  |  |  |  |  |  |  |  |
| 2010 | 14 (0.2) | 3 (0.2) | 11 (0.3) | 0.16 | <0.001 |  | 7 (0.2) | 3 (0.2) | 4 (0.2) | 0.04 | 0.99 |
| 2011 | 538 (8.9) | 132 (6.6) | 406 (10.1) |  |  |  | 261 (6.8) | 131 (6.8) | 130 (6.7) |  |  |
| 2012 | 455 (7.6) | 163 (8.2) | 292 (7.3) |  |  |  | 314 (8.1) | 154 (8.0) | 160 (8.3) |  |  |
| 2013 | 687 (11.4) | 220 (11.0) | 467 (11.6) |  |  |  | 424 (11.0) | 217 (11.2) | 207 (10.7) |  |  |
| 2014 | 675 (11.2) | 206 (10.3) | 469 (11.7) |  |  |  | 406 (10.5) | 201 (10.4) | 205 (10.6) |  |  |
| 2015 | 919 (15.3) | 300 (15.0) | 619 (15.4) |  |  |  | 595 (15.4) | 290 (15.0) | 305 (15.8) |  |  |
| 2016 | 920 (15.3) | 315 (15.8) | 605 (15.0) |  |  |  | 606 (15.7) | 305 (15.8) | 301 (15.6) |  |  |
| 2017 | 1077 (17.9) | 403 (20.2) | 674 (16.7) |  |  |  | 739 (19.1) | 379 (19.6) | 360 (18.7) |  |  |
| 2018 | 737 (12.2) | 256 (12.8) | 481 (12.0) |  |  |  | 508 (13.2) | 250 (13.0) | 258 (13.4) |  |  |

**Supplementary table 3:** Balance summary of covariates before and after matching on propensity scores.

*Data are presented as median (interquartile range).

BP, blood pressure; HR, heart rate; LVEF, left ventricular ejection fraction; CCI, Charlson Comorbidity Index; IHD, ischemic heart disease

## Supplementary Figures

**Supplementary figure 1:** Standardized difference of covariates before and after matching on propensity scores.


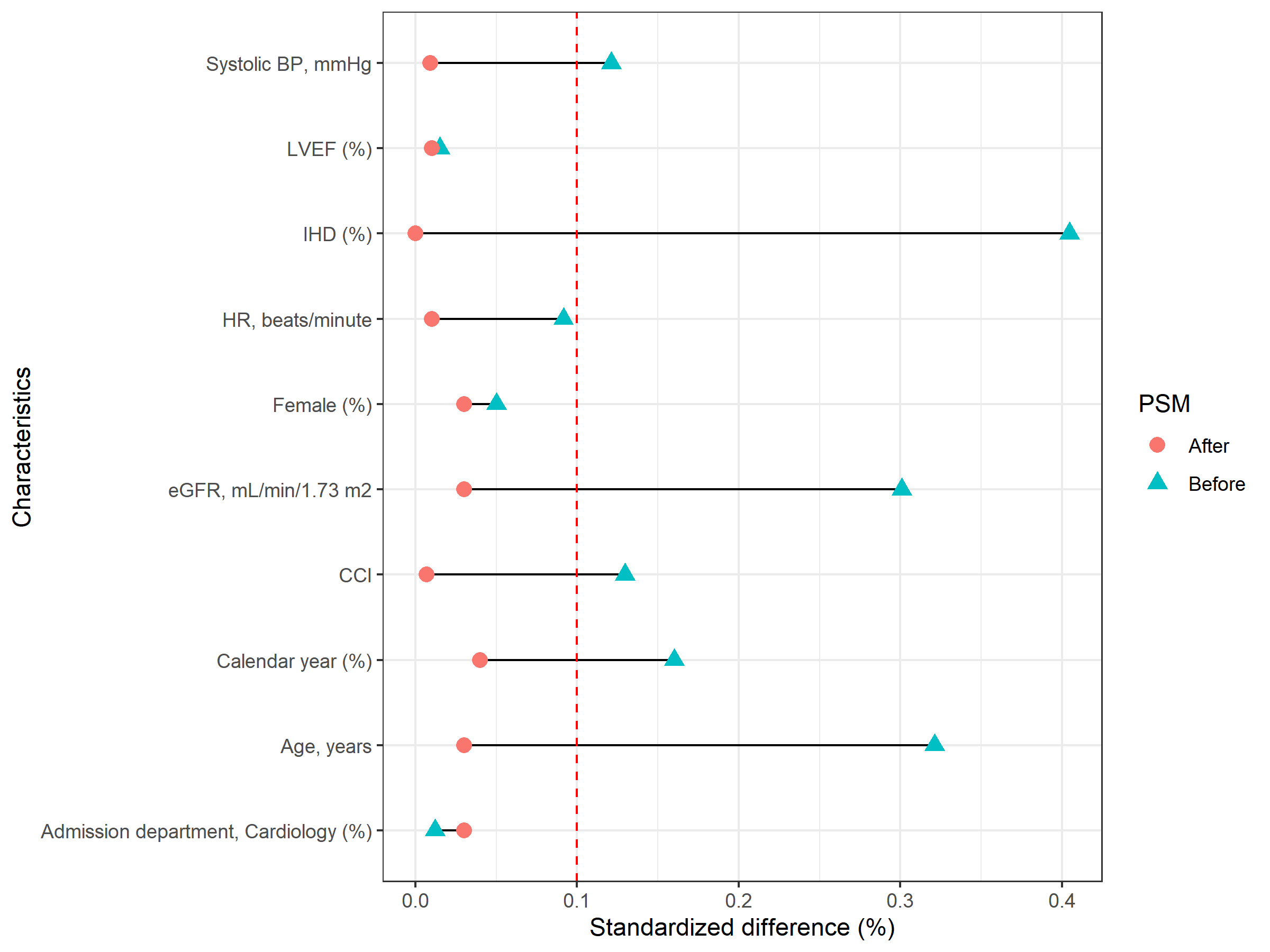


BP, blood pressure; HR, heart rate; LVEF, left ventricular ejection fraction; CCI, Charlson Comorbidity Index; IHD, ischemic heart disease; PSM, propensity score matching
